# Erratum for Baddal et al., Dual RNA-seq of Nontypeable *Haemophilus influenzae* and Host Cell Transcriptomes Reveals Novel Insights into Host-Pathogen Cross Talk

**DOI:** 10.1128/mBio.00373-16

**Published:** 2016-04-12

**Authors:** Buket Baddal, Alessandro Muzzi, Stefano Censini, Raffaele A. Calogero, Giulia Torricelli, Silvia Guidotti, Anna R. Taddei, Antonello Covacci, Mariagrazia Pizza, Rino Rappuoli, Marco Soriani, Alfredo Pezzicoli

**Affiliations:** a R&D Centre, GSK Vaccines, Siena, Italy; b Department of Molecular Biotechnology and Health Sciences, Molecular Biotechnology Center, University of Turin, Turin, Italy; c Center for High Instruments, Electron Microscopy Section, University of Tuscia, Viterbo, Italy

## ERRATUM

Volume 6, no. 6, doi:10.1128/mBio.01765-15, 2015. The transcriptomic data sets relative to host and nontypeable *Haemophilus influenzae* (NTHi) response were omitted from the supplemental material. They have been added to the supplemental material online. Gene modulation is expressed as log_2_ fold change at specific time points compared to the reference condition. The revised supplemental list should appear as below:

## SUPPLEMENTAL MATERIAL

Figure S1  Download Figure S1, PDF file, 0.8 MB

Figure S2  Download Figure S2, PDF file, 0.5 MB

Figure S3  Download Figure S3, PDF file, 0.4 MB

Figure S4  Download Figure S4, PDF file, 0.4 MB

Figure S5  Download Figure S5, PDF file, 0.4 MB

Table S1  Table S1, PDF file, 0.1 MB

Table S2  Table S2, PDF file, 0.2 MB

Table S3  Table S3, PDF file, 0.2 MB

Data Set S1 Download Data Set S1, XLSX file, 0.2 MB

Data Set S2 Download Data Set S2, XLSX file, 0.2 MB

