# Supplementary material for: Erratum for Baddal et al., Dual RNA-seq of Nontypeable Haemophilus influenzae and Host Cell Transcriptomes Reveals Novel Insights into Host-Pathogen Cross Talk
Source: mBio. 2016 Apr 12;7(2):e00373-16. doi: 10.1128/mBio.00373-16 (PMC4966755; doi:10.1128/mBio.00373-16)
Supplement: Figure S1 — Download [file mbo006152554sf1.pdf]

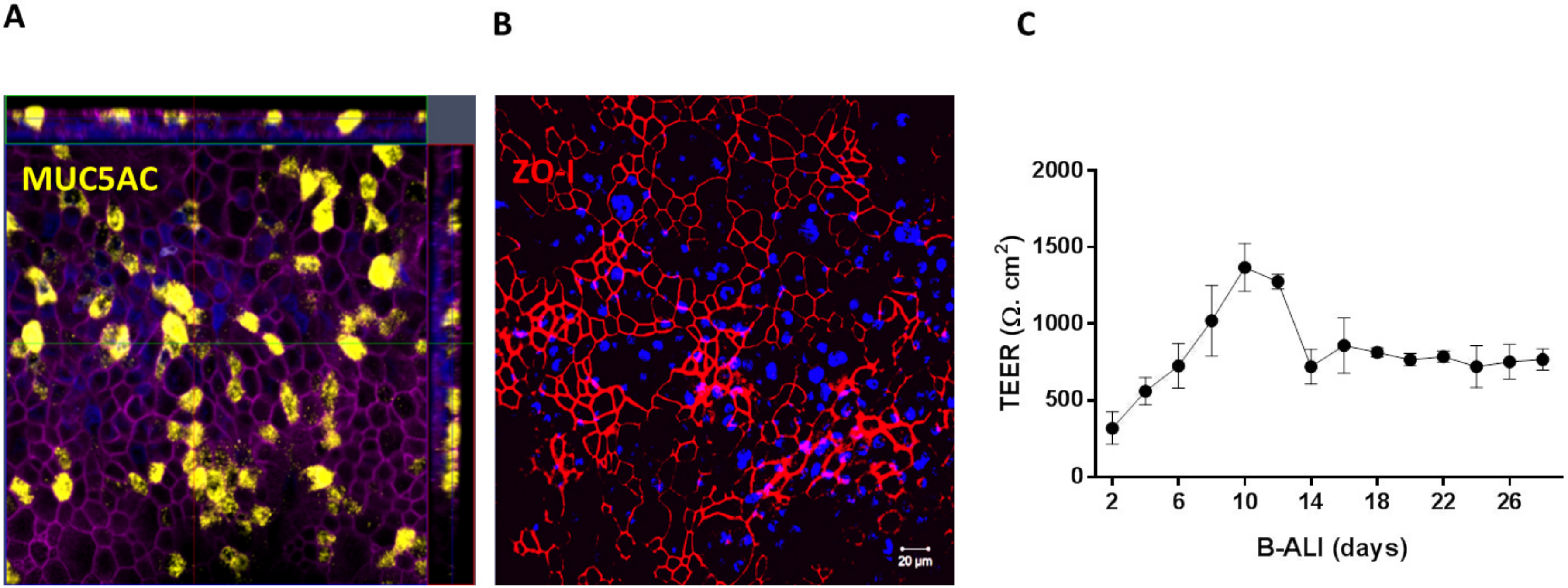

FIG S1 Characterization of WD-NHBE cells. (A) Orthogonal section of WD-NHBE cells stained for goblet cell marker MUC5AC (yellow); phalloidin (magenta); nuclei (blue) at 28 days of culture. (B) *En face* confocal image of tight junction marker ZO-I (red), nuclei were counterstained with Hoechst 33342 (blue). (Magnification: 40x.) (C) TER measurements throughout the differentiation schedule, values above 600 Ω representative of an effective epithelial barrier functioning.
