# Supplementary material for: Erratum for Baddal et al., Dual RNA-seq of Nontypeable Haemophilus influenzae and Host Cell Transcriptomes Reveals Novel Insights into Host-Pathogen Cross Talk
Source: mBio. 2016 Apr 12;7(2):e00373-16. doi: 10.1128/mBio.00373-16 (PMC4966755; doi:10.1128/mBio.00373-16)
Supplement: Figure S2 — Download [file mbo006152554sf2.pdf]

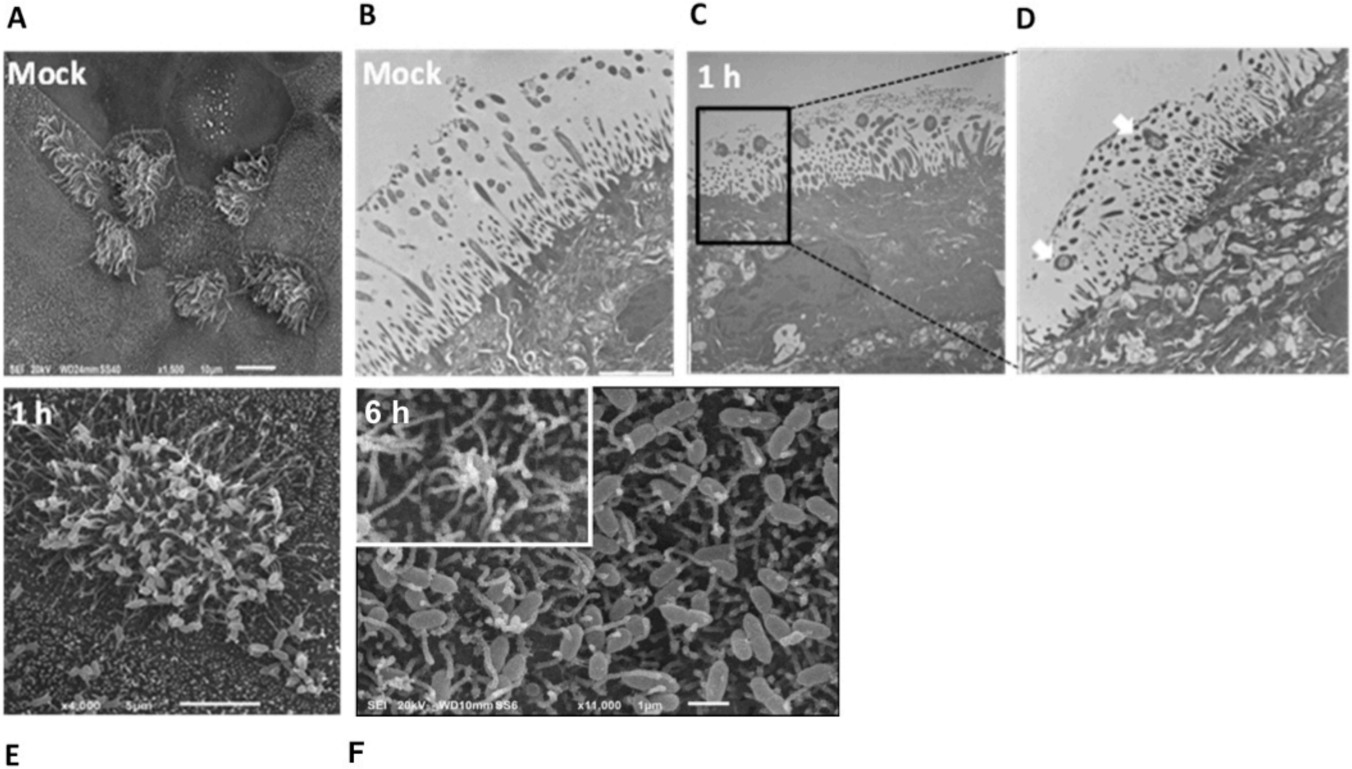

FIG S2 Electron microscopy examination of ciliated human bronchial epithelium during NTHi challenge (A) SEM and (B) TEM micrographs of epithelia prior infection. (C-D) TEM and (E) SEM micrographs of epithelia at 1 h post infection demonstrating NTHi ciliary binding. (F) SEM micrograph showing the morphological changes in infected host cell at 6 hpi as indicated by transcriptome signatures in IPA modules. The host cells extend microvilli and form protrusions around adhered bacteria. Scale: 1-10μm.
