# Supplementary material for: Erratum for Baddal et al., Dual RNA-seq of Nontypeable Haemophilus influenzae and Host Cell Transcriptomes Reveals Novel Insights into Host-Pathogen Cross Talk
Source: mBio. 2016 Apr 12;7(2):e00373-16. doi: 10.1128/mBio.00373-16 (PMC4966755; doi:10.1128/mBio.00373-16)
Supplement: Figure S3 — Download [file mbo006152554sf3.pdf]

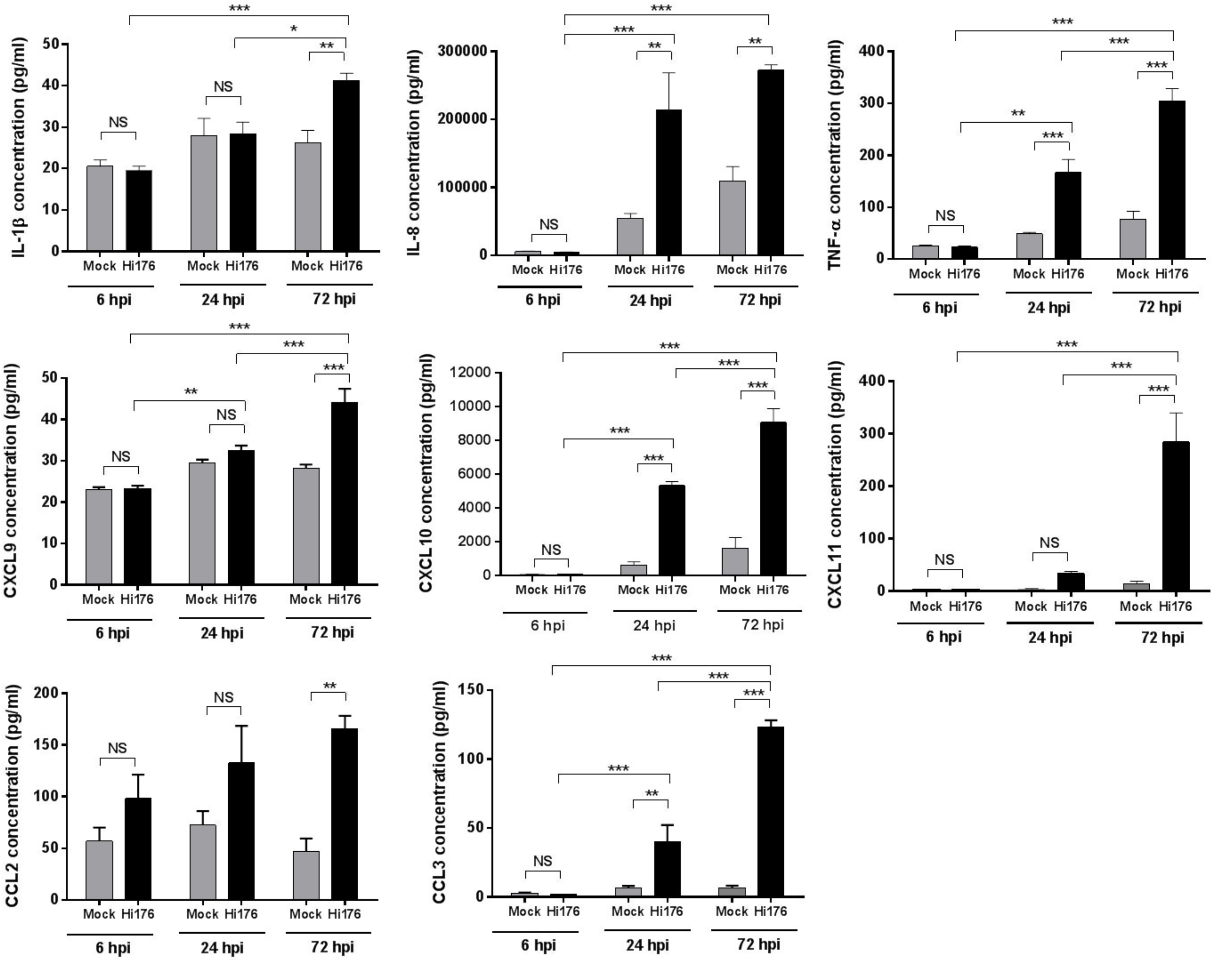

FIG S3 Basolateral cytokine/chemokine secretions induced following NTHi infection. WD-PBECs were infected as indicated in Fig. 1. Cytokine secretions in the basolateral medium of NTHi- and mock-infected cultures harvested at 6, 24, and 72 hpi were measured. Values are means + SEM. *P < 0.05, **P < 0.01, ***P < 0.001.
