# Supplementary material for: Erratum for Baddal et al., Dual RNA-seq of Nontypeable Haemophilus influenzae and Host Cell Transcriptomes Reveals Novel Insights into Host-Pathogen Cross Talk
Source: mBio. 2016 Apr 12;7(2):e00373-16. doi: 10.1128/mBio.00373-16 (PMC4966755; doi:10.1128/mBio.00373-16)
Supplement: Figure S4 — Download [file mbo006152554sf4.pdf]

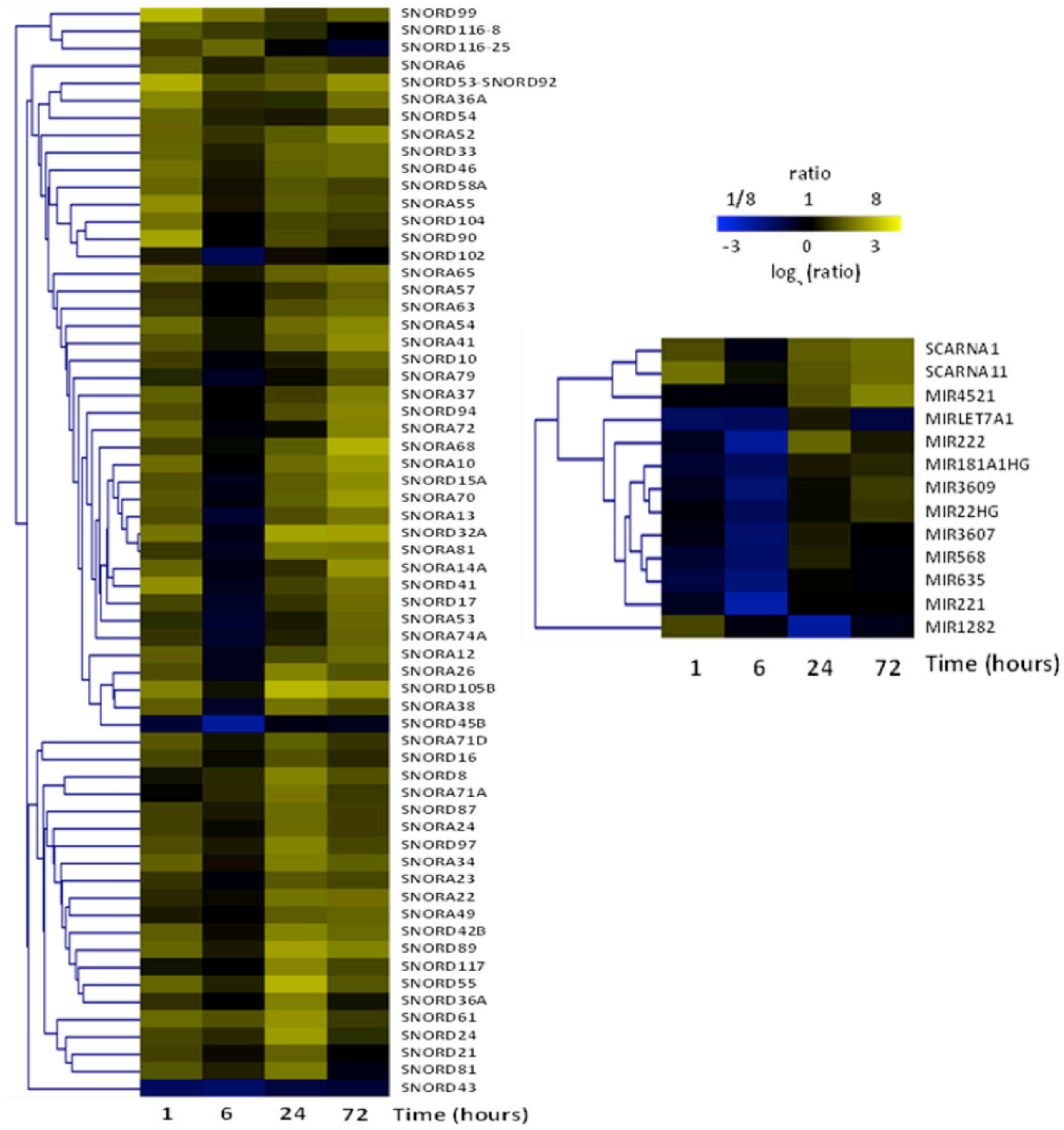

FIG S4 Heat-map profiles of novel snoRNAs, microRNAs and scaRNAs identified to be differentially expressed during NTHi infection in host cells.
