# Supplementary material for: Erratum for Baddal et al., Dual RNA-seq of Nontypeable Haemophilus influenzae and Host Cell Transcriptomes Reveals Novel Insights into Host-Pathogen Cross Talk
Source: mBio. 2016 Apr 12;7(2):e00373-16. doi: 10.1128/mBio.00373-16 (PMC4966755; doi:10.1128/mBio.00373-16)
Supplement: Figure S5 — Download [file mbo006152554sf5.pdf]

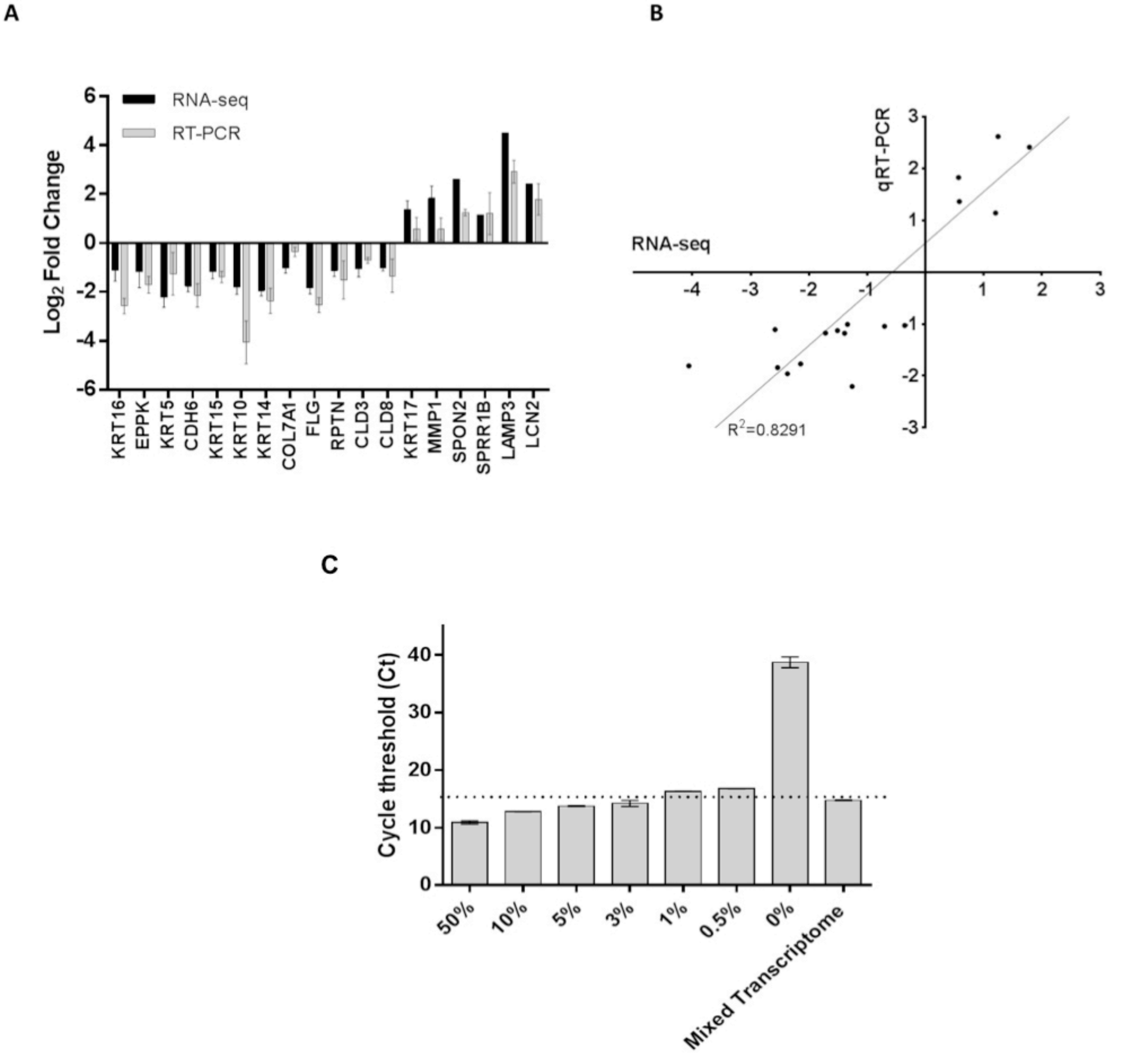

FIG S5 Validation of comparative RNA-seq analysis. (A) qRT-PCR analysis of representative genes identified by RNA-seq. The x-axis represents individual genes and the y-axis fold-change in expression by RNA-seq (black bars) or qRT-PCR (gray bars). (B) Correlation coefficient (R) between two data-sets. (C) qRT-PCR experiments to assess bacterial RNA contents in mixed transcriptome sample. The presence of bacterial RNA in the mixed transcriptome sample (1 h post infection) was examined by qRT-PCR using artificially mixed transcriptome samples at several bacterial RNA concentrations (0, 0.5, 1, 3, 5, 10 and 50%). Cycle threshold (Ct) values represent the point at which the amount of the amplified 16S reached a fixed threshold. High Ct values indicate low abundance of bacterial RNA content. The means and standard errors of Ct values of three technical replicates are shown for each sample.
