# Supplementary material for: Erratum for Baddal et al., Dual RNA-seq of Nontypeable Haemophilus influenzae and Host Cell Transcriptomes Reveals Novel Insights into Host-Pathogen Cross Talk
Source: mBio. 2016 Apr 12;7(2):e00373-16. doi: 10.1128/mBio.00373-16 (PMC4966755; doi:10.1128/mBio.00373-16)
Supplement: Table S1 — , PDF file, 0.1 MB [file mbo006152554st1.pdf]

TABLE S1 Summary of Illumina RNA-seq mapping data. Total number reads obtained and total number of reads mapping to the hg19 and NTHi reference genomes with 100% accuracy are shown. Percentages of mapping analysis are shown in parenthesis.

Library	Total # of reads	Total # of reads mapped to human genome	Total # of reads mapped to NTHi genome
t1 uninfected NHBE R1	94,635,010	51,123,742 (54.02%)	-
t1 uninfected NHBE R2	67,129,188	52,591,230 (78.34%)	-
t1 uninfected NHBE R3	89,824,350	73,530,786 (81.86%)	-
t1 infected NHBE R1	75,860,270	33,755,564 (44.50%)	1,083,698 (1.43%)
t1 infected NHBE R2	158,135,300	86,126,428 (54.46%)	2,273,776 (1.44%)
t1 infected NHBE R3	152,579,366	77,471,824 (50.77%)	2,208,934 (1.45%)
t6 infected NHBE R1	175,966,596	104,761,274 (59.53%)	1,705,098 (0.97%)
t6 infected NHBE R2	146,780,238	92,029,526 (62.70%)	1,707,726 (1.16%)
t6 infected NHBE R3	174,955,950	97,498,086 (55.73%)	1,687,994 (0.96%)
t24 uninfected NHBE R1	110,051,896	90,732,072 (82.44%)	-
t24 uninfected NHBE R2	93,282,492	78,653,468 (84.32%)	-
t24 uninfected NHBE R3	97,111,032	81,928,398 (84.37%)	-
t24 infected NHBE R1	114,759,310	48,929,464 (42.64%)	228,867 (0.20%)
t24 infected NHBE R2	115,486,974	86,939,972 (73.55%)	695,776 (0.60%)
t24 infected NHBE R3	94,635,372	69,706,936 (73.66%)	625,927 (0.66%)
t72 uninfected NHBE R1	100,592,622	76,650,932 (76.20%)	-
t72 uninfected NHBE R2	104,098,524	87,174,036 (83.74%)	-
t72 uninfected NHBE R3	99,218,056	79,030,560 (79.65%)	-
t72 infected NHBE R1	83,329,496	56,060,568 (67.28%)	476,744 (0.57%)
t72 infected NHBE R2	117,197,758	90,650,622 (77.35%)	912,480 (0.78%)
t72 infected NHBE R3	106,284,334	73,401,304 (69.06%)	619,618 (0.58%)
t1c Hi176 control R1	100,496,004	181,430 (0.18%)	29,983,584 (29.84%)
t1c Hi176 control R2	61,902,856	542 (0.00%)	18,119,799 (29,27%)
t1c Hi176 control R3	130,037,454	844,358 (0.65%)	50,244,630 (38,64%)
