# Supplementary material for: Erratum for Baddal et al., Dual RNA-seq of Nontypeable Haemophilus influenzae and Host Cell Transcriptomes Reveals Novel Insights into Host-Pathogen Cross Talk
Source: mBio. 2016 Apr 12;7(2):e00373-16. doi: 10.1128/mBio.00373-16 (PMC4966755; doi:10.1128/mBio.00373-16)
Supplement: Table S2 — , PDF file, 0.2 MB [file mbo006152554st2.pdf]

TABLE S2 Selected paired Ingenuity Pathway Analysis (IPA) of host functions

Top molecular and cellular functions summary

Time-point	Top Molecular and Cellular Functions*	Number of Molecules	p-value
**1h**	Cell Death and Survival	11	2.99E-05
	Carbohydrate Metabolism	10	6.33E-05
	Molecular Transport	10	6.33E-05
	Small Molecular Biochemistry	10	6.33 E-05
	Gene Expression	0	6.62E-05
**6 h**	Cell Cycle	66	2.77E-09
	Cellular Development	120	3.96E-09
	Cellular Growth and Proliferation	130	3.96E-09
	Cellular Movement	90	2.40E-08
	Cell Death and Survival	123	3.26E-08
**24 h**	Cell-To-Cell Signaling and Interaction	20	6.68E-06
	Cellular Movement	30	6.68E-06
	Cell Signaling	13	3.37E-05
	Carbohydrate Metabolism	10	1.19E-04
	Small Molecule Biochemistry	23	1.19E-04
**72 h**	Cellular Movement	70	3.20E-08
	Cell Death and Survival	89	5.78E-08
	Cell-To-Cell Signaling and Interaction	52	1.81E-07
	Cellular Growth and Proliferation	104	2.63E-07
	Post-Translational Modification	4	8.41E-06

*The functional Analysis of a Network identified biological functions that were most significant to the molecules in the network using a right-tailed Fisher's exact test.

Top canonical pathways summary

Time-point	Top Canonical Pathways*	Ratio	p-value
1h	Factors Promoting Cardiogenesis in Vertebrates	3/92	1.54E-04
	Human Embryonic Stem Cell Pluripotency	3/134	4.67E-04
	Basal Cell Carcinoma Signaling	2/72	3.06E-03
	Glutathione Biosynthesis	1/3	3.44E-03
	TR/RXR Activation	2/85	4.24E-03
6 h	Erb Signaling	8/86	1.33E-04
	G-Protein Coupled Receptor Signaling	14/256	1.81E-04
	Trombopoietin Signaling	6/55	3.90E-04
	Acute Myeloid Leukemia Signaling	7/77	4.02E-04
	ERK/MAPK Signaling	11/187	4.79E04
24 h	Pathogenesis of Multiple Scleroris	3/9	1.59E-05
	Differential Regulation of Cytokine Production by IL-17A and IL-17F	3/23	3.16E-04
	IL-17A Signaling in Gastric Cells	3/25	4.07E-04
	Granulocyte Adhesion and Diapedesis	6/177	6.07E-04
	Agranulocyte Adhesion and Diapedesis	6/189	8.55E-04
72 h	Granulocyte Adhesion and Diapedesis	13/177	5.44E-07
	Interferon Signaling	6/36	6.21E-06
	Role of IL-17A in Psoriasis	4/13	1.78E-05
	Airway Pathology in COPD	3/8	1.14E-04
	Pathogenesis of Multiple Sclerosis	3/9	1.70E-04

*Canonical Pathway Analysis identified pathways from the IPA library that were most significant to the data set. Significance of the association was measured in two ways: (1) as the ratio of the number of molecules from the focus gene set that map to the pathway to the total number of molecules that map to the canonical pathway and (2) using Fisher's exact test.

Summary of Cellular Assembly and Organization module (cell morphology). Activation scores across cytostructural components including multiple extracellular matrix proteins, laminins, collagens and cytoskeletal proteins indicate modulation of host cytoskeletal architecture during NTHi infections.

**Enriched host functions at 6 hpi**	**Activation score**	**P value**
Formation of fibronectin matrix	1.72	1.18E-03
Formation of lamellipodia	1.65	1.40E-04
Ruffling	1.34	2.57E-03
Disruption of cytoskeleton	1.34	1.03E-03
Formation of cellular protrusions	0.83	1.88E-03
Organization of cytoskeleton	0.51	2.15E03
Extension of lamellipodia	0.45	7.80E-05
Formation of actin filaments	0.44	2.65E-04
