# Supplementary material for: Erratum for Baddal et al., Dual RNA-seq of Nontypeable Haemophilus influenzae and Host Cell Transcriptomes Reveals Novel Insights into Host-Pathogen Cross Talk
Source: mBio. 2016 Apr 12;7(2):e00373-16. doi: 10.1128/mBio.00373-16 (PMC4966755; doi:10.1128/mBio.00373-16)
Supplement: Table S3 — , PDF file, 0.2 MB [file mbo006152554st3.pdf]

TABLE S3 Oligonucleotides and antibodies used in this study

Primer Name	Primer Sequence 5'-3'
ADD3 F	TGACCATACAGGATTCAGTC
ADD3 R	CCACATTTCATGGAGGATAC
CDH6 F	GTCACAGCCCAAGATCCAGATGCT
CDH6 R	GTGCCATAGCAGTGTTTCTCGGTCA
COL7A1 F	ATGACCTTGGCATTATCTTG
COL7A1 R	TGAATATGTCACCTCTCAAGG
EPPK F	GGTTCAGAGGCCAGAAACCAACAC
EPPK R	CTCGATCAACTCTAAGATGAGCTGCGC
FLG F	AATTTCGGCAAATCCTGAAG
FLG R	CTTGAGCCAACTTGAATACC
GAPDH F	CATGGCACCGTCAAGGCTGAGAA
GAPDH R	CAGTGGACTCCACGACGTACTCA
KRT 5 F	GCATCACCGTTCCTGGGTAACAG
KRT 5 R	CGCTCCGGAAGGACACACTT
KRT 6B F	TCAGCACTCAGACATGCGAATGTCC
KRT 6B R	GAGGACTCCTCATCTGCAGCTGG
KRT 10 F	GGACATAGAACTACAGTCCC
KRT 10 R	GTATTCAGTATTCTGGCACTC
KRT 14 F	GAGATGTCAATGTGGAGATG
KRT 14 R	GTCTTGGTGAAGAACCATTC
KRT 15 F	AGGTGTGCAGGCAGCTGTGTTTG
KRT 15 R	AGAGGGTGTTGTGGGACCTCGT
KRT 16 F	CATCGAGGACCTGAGGAACAAGGT
KRT 16 R	GACCCTCTGCTACCACTTCCCTG
KRT 17 F	GTGAGATCAATGTGGAGATG
KRT 17 R	GTCTTGCTGAAGAACCAATC
LAMP3 F	CAGAAAAGTCCACATAACCC
LAMP3 R	AACACATGAGGAAAGTCAAC
LCN2 F	GGAAAAAGAAGTGTGACTACTG
LCN2 R	GTAACTCTTAATGTTGCCCAG
MMP1 F	GCTAACAAATACTGGAGGTATG
MMP1 R	ATCAACTTTGTGGCCAATTC
RPTN F	ATCCTCCAGAGACCAAATG
RPTN R	TCTAGCTTATGATAGCAGGC
SPON2 F	CTTGAGAAGTGAATAAATGGGG
SPON2 R	TTTGTGAGCAACAGAGATAG
SPRR1B F	TATTCCTCTCTTCACACCAG
SPRR1B R	TCCTTGGTTTTGGGGATG
TUBA1A F	AGATGCTGCCAATAACTATG
TUBA1A R	CTTGCCATAATCAACTGAGAG
16S F	GGTGCTGCATGGCTGTCGTC
16S R	CACCTCGCAGCTTCGCTTCC

Antibody/Fluorescent dye	Dilution	Company	Cat No.
Rabbit anti-NTHi serum	IF 1:5000	In house	-
B-tubulin IV	IF 1:200	Sigma	T7941
MUC5AC	IF 1:200	Millipore	MAB2011
ZO-I	IF 1:200	Invitrogen	61-7300
Pan cytokeratin	IF 1:200	Invitrogen	18-0132
KRT10	WB 1:5000	Abcam	Ab76318
K-cadherin	WB 1:1000	Thermo Scientific	PA5-11456
Claudin 3	WB 1:500	Abcam	Ab52231
Adducin gamma	WB 1:500	Abcam	Ab135868
GAPDH	WB 1:2000	Life Technologies	AM4300
Anti-Mouse HRP	1:2500	Dako	P0161
Anti-Rabbit HRP	1:2500	Dako	P0448
Alexa Fluor 568 anti mouse	1:1000	Life Technologies	A11004
Alexa Fluor 568 anti rabbit	1:1000	Life Technologies	A11011
Alexa Fluor 488 anti mouse	1:1000	Life Technologies	A11079
Alexa Fluor 647 phalloidin	1:400	Life Technologies	A22287
Hoechst 33342	1:10000	Life Technologies	H3570
